# The Safe Gluteoplasty: Anatomic Landmarks to Predict the Superior and Inferior Gluteal Veins

**Published:** 2019-03-19

**Authors:** Claude Muresan, Jared M. Davis, Andrea R. Hiller, Brittany E. Patterson, Christina N. Kapsalis, Meghan F. Ford, Eric W. Anderson, Swapnil D. Kachare, Ron Hazani, Bradon J. Wilhelmi

**Affiliations:** ^a^Division of Plastic Surgery, General Surgery Department, University of Louisville, Louisville, Ky; ^b^University of Louisville School of Medicine, Louisville, Ky. Dr Hazani is in private practice, Beverly Hills, Calif

**Keywords:** gluteoplasty, lipoinjection, gluteal veins, pulmonary fat embolism, anatomic landmarks

## Abstract

**Objective:** The increase in demand for gluteal fat grafting seen in recent years in the United States has not been met with an equal gain in knowledge of the perils of this anatomic territory. The purpose of this study was to identify anatomic landmarks that can be readily used by surgeons to identify the takeoff of the superior and inferior gluteal veins. **Method:** Six fresh cadaveric gluteal specimens were dissected at the University of Louisville anatomy laboratory. A question mark incision was made for exposure, followed by identification of the sciatic nerve in the proximal thigh. This was traced retrograde to the sciatic forearm. The piriformis muscle was identified dividing the foreman into superior and inferior portions, which corresponded to the takeoff of the superior and inferior gluteal vessels, respectively. The distance of the gluteal vessels from the one-third point of a line from the mid-sacrum to the greater trochanter was measured. **Result:** Our cadaveric dissection series demonstrated that the superior and inferior gluteal veins were on average 3.28 cm (2-5.9 cm) and 1.25 cm (0-3.5 cm) away from the point one third the distance from the mid-sacral border to the greater trochanter. **Conclusion:** The mid-sacrum and the trochanter of the femur are the anatomic landmarks used to identify the large gluteal vein trunks. Understanding the location and trajectory of these deep gluteal structures with use of readily identifiable landmarks may assist surgeons in avoiding inadvertent injection of fat to these veins during fat grafting.

Gluteal augmentation with fat grafting is a procedure that has seen a dramatic increase in popularity in recent years, ranking 10th of all surgical procedures performed by members of the International Society of Aesthetic Plastic Surgery in 2016.^1^ Similarly in 2016, the United States saw a 26% increase, more than 18,000 operations, from the prior year.^2^ The aesthetic appeal of a small waist and full buttocks is far from a novel concept, as these features have been idealized by numerous cultures and ethnicities due to their association with female reproductive potential and physical well-being.^3^^,^^4^ According to Wong et al,^5^ the new ideal waist-to-hip ratio is 0.6 and 0.65 compared with 0.7, which represents a shift in preference to a “curvier” silhouette. Increased interest in gluteal augmentation has been fueled by a greater focus on body image due to modern fashion trends, such as thong swim suits, and popular media that sensationalize women with ample buttocks as the standard for beauty.^6^^,^^7^ While patient satisfaction with gluteal augmentation is reportedly very high, the procedure is not without risks.^8^^,^^9^

As the number of gluteal augmentation procedures performed has increased, reports have surfaced linking a number of patient fatalities directly to gluteal fat grafting.^10^^-^12 The first case report on fatal pulmonary fat embolism (PFE) due to gluteal fat grafting was published in the *Journal of Forensic Sciences* in 2015.^12^ However, this complication did not gain widespread attention until Cárdenas-Camarena et al^10^ published a retrospective study reporting 22 deaths in Mexico and Columbia over a 15-year period attributed to PFE after gluteal fat grafting. Because of increasing awareness of significant patient morbidity and mortality, the Aesthetic Surgery Education and Research Foundation (ASERF) formed a task force to examine the risk of PFE associated with gluteal fat grafting. Their data suggest the annual mortality rate from PFE secondary to gluteal fat grafting to be 1:3448, although they estimate the actual mortality rate may be as high as 1:2351 or greater.^11^

To avoid PFE during gluteal fat augmentation, it is imperative that plastic surgeons have a deep understanding of both the technique and gluteal anatomy, particularly gluteal vein trajectory.^3^^,^^13^ There is still much debate regarding the safest technique for gluteal augmentation.^13^^,^^14^ While there have been descriptions of large “danger areas” to avoid during gluteal augmentation, these zones often encompass 40% or more of the gluteal surface, making enhancement challenging, if not impossible, with complete avoidances of the areas. Furthermore, having a danger zone so large defeats the purpose of alerting the operator to a precise area peril.^9^^,^^15^ Our study aims to identify simple and reproducible exterior anatomic landmarks for identification of the superior and inferior gluteal veins. With reliable landmarks, surgeons can proceed with an increased awareness of this precise “danger zone” and reduce the risk of inadvertent injection of fat into the gluteal vessels during buttock augmentation with fat grafting.

## METHODS

**Figure 1:**
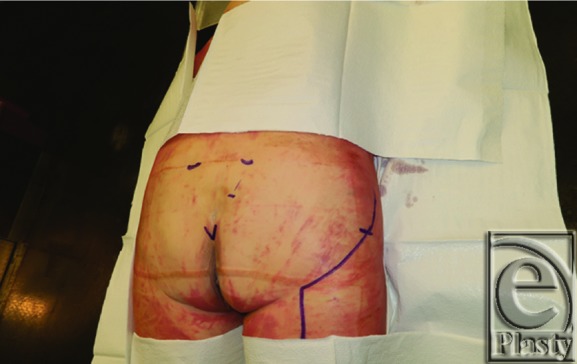
Preoperative markings showing greater trochanter, posterior superior iliac spine, coccygeal tip, mid-sacral border, and questions mark incision.

**Figure 2:**
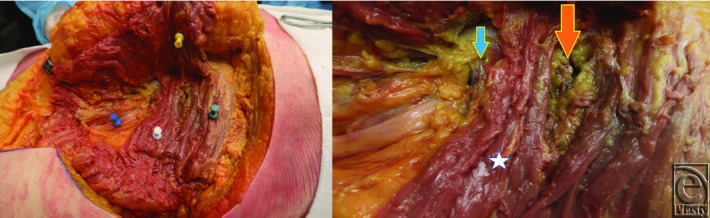
(Left) Dissection demonstrating sciatic nerve (blue push pin), retracted gluteus maximus (yellow push pin), piriformis muscle (white push pin), and gluteus medius (green push pin) with underlying superior gemellus muscle. (Right) Close-up view of dissection showing the superior gluteal vessel (large orange arrow), inferior gluteal vessel (small blue arrow), and piriformis muscle (white star).

Six cadaveric gluteal specimens were dissected at the University of Louisville fresh tissue laboratory. Each cadaver was female and less than 24 hours old. One specimen was discarded because of a posterior hip dislocation. All dissections were carried out in a stepwise fashion, with the cadaver placed at 0°, prone position. Prior to incision, the following surface landmarks were marked: coccygeal tip, posterior superior iliac spine (PSIS), mid-sacral border (measured halfway between the coccygeal tip and the PSIS), and the greater trochanter (Fig 1). A question mark incision was made for exposure and carried down to the level of the gluteus maximus muscle. Next, the sciatic nerve was first identified distally in the upper posterior thigh and then traced retrograde to the sciatic foreman. The piriformis muscle was identified dividing the foreman into superior and inferior portions, which corresponded to the takeoff points of the superior (SGV) and inferior gluteal vessels (IGV), respectively (Fig 2).

**Figure 3:**
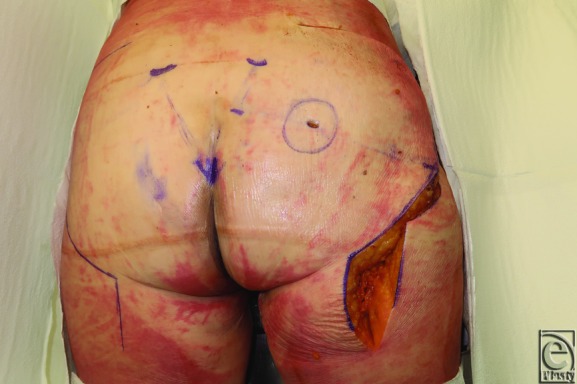
Preoperative marking showing a line drawn from the mid-sacral border to the greater trochanter. One-third point is incised, and a circle with a radius of 3.3 cm drawn around the one-third point.

**Figure 4:**
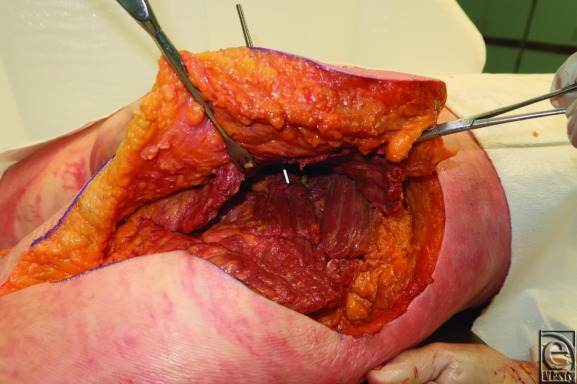
Round knife handle inserted through the one-third point.

Following identification of the gluteal vessels, a line was drawn on the skin from the mid-sacral border to the PSIS. We then measured and marked a point one third the distance from the mid-sacral border to the trochanter (Fig 3). The external line on the skin surface corresponded to the trajectory of the piriformis muscle. A pinpoint incision was made at the one-third distance point and a round knife handle was inserted through the incision (Fig 4). The handle was used to create a direct trajectory from the external one-third point to the gluteal vessel below. The distance of the SGV and IGV veins to the one-third point was measured.

Following completion of this primary measurement, several other measurements were recorded. This included the distance of the superior and inferior vessels from the PSIS, the intergluteal vessel distance, and the distance of the gluteal vessels from the sacral border.

## RESULTS

A line drawn from the mid-sacrum to the trochanter marks the trajectory of the piriformis muscle. The superior and inferior gluteal veins emerge from the superior and inferior aspects of this muscle's insertion to the sacrum, respectively. Our cadaveric dissection series demonstrated that, on average, the distance of the superior and inferior gluteal veins from the one-third point was 3.3 cm (2-5.9 cm) and 1.25 cm (0-3.5 cm), respectively (Table 1). In other words, the main trunks of the superior and inferior gluteal veins are found on average within a 3.3-cm radius circle located one third the distance from the mid-sacral border to the greater trochanter. The average distance of the line from mid-sacral border to the greater trochanter was 16.7 cm (14-19 cm), and the average distance of the one-third point from the mid-sacral border was 5.5 cm (4.6-6.3 cm). On average, the superior gluteal vein is approximately 1 cm closer to the sacral border than the inferior gluteal veins (1.9 vs 2.8 cm). The superior gluteal vein is on average 4.3 cm (3.5-5 cm) above the inferior gluteal vein. The average distance of the SGV and IGV from the PSIS was 8.1 cm (6.5-9 cm) and 12.4 cm (11.5-14 cm), respectively. In the third dissection, 2 IGVs were identified draining into the internal iliac via a common trunk (Table 1). Each vein's distance was recorded separately and used independently for calculation of averages.

## DISCUSSION

In 1999, Cárdenas-Camarena et al^16^ first described the technique of combining liposuction and lipoinjection in the gluteal region to improve gluteal contour. Compared with implants, autologous fat grafting has the unique advantage of reshaping the buttocks and allowing for specific volume adjustments where necessary.^13^ The ability to perform targeted volume replacement, along with the minimally invasiveness of the operation, has caused gluteal fat grafting to become an increasingly popular procedure in recent years.^13^ Despite initial enthusiasm for the procedure, shortly thereafter several articles, including a landmark article in 2015 by Cárdenas-Camarena et al,^10^ directly attributed gluteal fat grafting to patient mortality.^10^^-^12 Autopsy reports and photographs in the Cárdenas-Camarena et al article demonstrated both micro- and macroscopic fat deposits in patients gluteal veins, vena cava, pulmonary arteries, and ventricles consistent with fat emboli as a direct cause of death. The exact mechanism of introduction of fat into systemic circulation is unknown; however, it is thought to be caused by either direct cannulation of the large gluteal vessels or the creation of a pressure gradient, due to lack of particle dispersion over several layers of tissues, and subsequent flow of fat into the low-pressure veins.^10^^,^^17^^,^^18^ As a result of an increasing number of reports of fatalities associated with gluteal fat grafting, the ASERF established a task force to objectively assess the number of fatal and nonfatal pulmonary fat emboli associated with the procedure. The preliminary results were shocking, suggesting an estimated mortality rate of 1 in 1253 or greater.

As a result of this high risk, authors have begun to describe techniques and anatomic safe zones in an attempt to improve patient safety and reduce mortality.^9^^-^11^,^^15^^,^^17^^,^^19^^,^^20^ The Villanueva et al^15^ article, “Staying Safe During Gluteal Fat Transplantation,” describes 4 key principles aimed to reduce PFE: use of a large-bore cannula (≥4 mm) to minimize the risk of deep muscle penetration and venous injury, continuous cannula motion to promote layered dispersal of fat and prevent inadvertent continuous injection into underlying vessels, staying subcutaneous to likewise avoid injury to the deeper veins, and avoiding overfilling in order to decrease the chance of creating a pressure gradient.^15^ The Ramos-Gallardo et al^17^ cadaveric study shed light on safe cannula angle to avoid insertion into the gluteal vessels. The authors concluded that when injecting at approximately 10 cm from the intergluteal crease and 6 cm from the upper border of the gluteal muscle, holding the cannula at an angle of 30° instead of 45° allowed fat injection without passage into deeper structures.^17^ Both these authors should be commended, as their timely technical modifications have helped surgeons minimize the risk of inadvertent gluteal vein injury, thus improving patient safety.

Likewise, to further improve patient safety, several authors have described anatomic “danger areas” of the gluteus to heighten awareness of the underlying gluteal vessels, which are near.^9^^,^^15^^,^^17^ The gluteal veins are found in the gluteus medius-minimus compartment. This compartment is formed superiorly by the deep gluteal fascia, laterally by the iliotibial tract, and inferiorly or deep by the ilium. The SGV exits superior to the piriformis, near the muscles insertion to the sacrum, whereas the IGV exits beneath the inferior edge of the piriformis, above the superior gemellus.^3^ Villanueva et al^15^ defined the “danger triangle” as bounded inferior laterally by the greater trochanter, inferior medially by the ischial tuberosity, and apex formed by the PSIS. Villanueva et al^15^ also adopted use of this triangle and emphasized that only superficial fat grafting be performed in this area. Rosique et al^8^ delineate a pyramidal “danger zone,” with its height along the intergluteal crease and its base following the medial two thirds of each gluteal fold. Similar to the previous authors, Rosique et al stressed avoidance of deep muscular fat injection while in this danger zone. We agree with all these authors that gluteal fat injection should be kept superficial in the subcutaneous tissues while in any of these danger zones and, furthermore, one might consider keeping all fat injection superficial, especially if one believes in the pressure gradient mechanism of PFE. Despite one's best attempts to stay superficial by using all the aforementioned techniques, there are times when deep penetration of the muscle inadvertently can occur. It is during this time that the surgeon must have precise knowledge as to exactly where the SGV and the IGV are located. All the danger zones described are relatively large areas encompassing 40% to 50% of the hemigluteal surface. They fail to provide this critical information to the operator.

Our study's purpose was to device a simple and reproducible technique that can be utilized to identify the specific location of the gluteal veins using external landmarks. Having precise knowledge of where the gluteal veins are located heightens the surgeon's awareness of when to be vigilant and allows for increased ability for fat augmentation because of a smaller, more defined danger zone. Our study demonstrated that these landmarks are the mid-sacral border, located halfway between the coccyx tip and the PSIS, and the greater trochanter. Drawing a line connecting these 2 landmarks and subsequently measuring one third the distance from the sacral border to the greater trochanter enables precise identification of the underlying gluteal vessels (Fig 3). As our data demonstrated that the SGV and the IGV will be on average 3.3 and 1.25 cm from this point, respectively. Having surgeons aware of this simple, and reproducible, technique for identification of the underlying gluteal vessels, we hope will help increase safety by heightening awareness to stay superficial at this area while enabling a complete augmentation.

Gluteal fat grafting will remain in the spotlight for years to come as surgeons continue to investigate techniques to improve safety. To avoid serious complications, it is imperative that surgeons have a firm understanding of the gluteal anatomy, especially of the underlying gluteal veins. This topic deserves more attention, and further studies are needed on outcomes as surgeons begin to implement recommendations outlined in recent literature aimed at improving patient safety during gluteal augmentation with fat grafting.

## Figures and Tables

**Table 1:** Distance between different anatomic locations and one-third reference point

	Dissection No.					
	1	2	3	4	5	Average
PSIS to SGV	9	8	8	6.5	9	8.1
PSIS to IGV	12.5	12	12, 12	11.5	14	12.4
SGV to IGV	3.5	4	4, 4	5	5	4.3
SB to IGV	3.5	3	3, 3.5	2	2.5	2.8
SB to SGV	2	2	2.5	1	3	1.9
Troch to Mid-SB	18.5	18	14	14	19	16.7
One-third distance mid-SB to Troch	6.2	6	4.6	4.6	6.33	5.5
SGV to one-third point	2	2	2	4.5	5.9	3.3
IGV to one-third point	2	0	3.5, 1.5	0	0.5	1.25

*PSIS indicates posterior superior iliac spine; SGV, superior gluteal vein; IGV, inferior gluteal vein; Troch, greater trochanter; and Mid-SB, mid-sacral border.
